# A Non-Death Role of the Yeast Metacaspase: Yca1p Alters Cell Cycle Dynamics

**DOI:** 10.1371/journal.pone.0002956

**Published:** 2008-08-13

**Authors:** Robin E. C. Lee, Lawrence G. Puente, Mads Kærn, Lynn A. Megeney

**Affiliations:** 1 Regenerative Medicine Program, Ottawa Health Research Institute, Sprott Centre for Stem Cell Research, Ottawa, Canada; 2 Department of Cellular and Molecular Medicine, University of Ottawa, Ottawa, Canada; 3 Ottawa Institute of Systems Biology, University of Ottawa, Ottawa, Canada; 4 Department of Physics, University of Ottawa, Ottawa, Canada; National Institutes of Health, United States of America

## Abstract

Caspase proteases are a conserved protein family predominantly known for engaging and executing apoptotic cell death. Nevertheless, in higher eukaryotes, caspases also influence a variety of cell behaviors including differentiation, proliferation and growth control. *S. cerevisiae* expresses a primordial caspase, *yca1*, and exhibits apoptosis-like death under certain stresses; however, the benefit of a dedicated death program to single cell organisms is controversial. In the absence of a clear rationale to justify the evolutionary retention of a death only pathway, we hypothesize that yca1 also influences non-apoptotic events. We report that genetic ablation and/or catalytic inactivation of Yca1p leads to a longer G1/S transition accompanied by slower growth in fermentation conditions. Downregulation of Yca1p proteolytic activity also results in failure to arrest during nocodazole treatment, indicating that Yca1p participates in the G2/M mitotic checkpoint. 20s proteasome activity and ROS staining of the *Δyca1* strain is indistinguishable from its isogenic control suggesting that putative regulation of the oxidative stress response by Yca1p does not instigate the cell cycle phenotype. Our results demonstrate multiple non-death roles for *yca1* in the cell cycle.

## Introduction

The timely and organized form of programmed cell death known as apoptosis has vital functions in the development and pathologies of complex metazoan life forms. Apoptosis is regulated by conserved signaling pathways that activate caspase proteases leading to target protein cleavage and cell death. Morphological features typical of apoptosis have been recognized in single cell organisms and structural homologues of the caspase family (metacaspases) have been identified [1]–[3]. Metacaspase proteases have since been characterized as apoptotic regulators in plants, fungi and protozoa supporting the concept of a widely conserved cell death pathway [4], [5].

A single metacaspase, YOR197w, was identified in the genome of *S. cerevisiae* and named Yeast Caspase 1, (Yca1p also known as MCA1). Yca1p demonstrates enzymatic peptidase activity analogous to mammalian caspase activity, and is inhibited in the presence of the fluoromethyl ketone (FMK) conjugated caspase-inhibitory substrate z-VAD-FMK [4]. In addition to canonical caspase substrate specificity Yca1p has secondary substrate specificity directed at arginine-lysine motifs [5]. Furthermore, Yca1p-mediated apoptosis has been observed in a variety of apoptogenic situations including senescence and following insults such as hydrogen peroxide (H_2_O_2_) and acetic acid [4], [6]–[9]. These findings have promoted the view of yeast as an evolutionarily distant model of metazoan apoptosis and numerous orthologous pathways have since been reported.

Despite the discovery of homologues to apoptotic proteins in yeast, there was limited consensus that yeast engaged a death program similar to that of multi-cellular metazoans. Arguments to rationalize apoptosis in yeast have included altruism in the presence of reactive oxygen species or killer toxins, a program to execute cells that fail to mate in response to pheromone and a mechanism for lateral gene transfer [6], [10]–[12]. In addition, the apoptotic pathway in yeast has been suggested to act as a mechanism to protect surrounding cells from the potentially damaging cellular constituents of dead or dying cells [13]. However, the validity of these hypotheses has yet to be fully explored and may not justify the development and retention of a biochemical pathway devoted solely to cell death. Without an incontrovertible justification for an apoptotic response in yeast, it is also reasonable to conjecture that proteins such as Yca1p may have initially developed or co-evolved other vital non-death functions [14], [15].

Caspase activity is an inductive signal for non-death functions such as cell cycle progression, proliferation, cell migration and differentiation in mammalian cell lines [14], [16]. Enzymatic processing of target proteins by caspase-3 at key time points promotes differentiation in mammalian precursor and progenitor cells [15], [17]. The enzymatic activity of caspases has also been associated with the proper activation and release from the mitotic checkpoint. In HeLa cells, disruption of microtubule dynamics results in caspase mediated cleavage of the mitotic checkpoint protein BubR1p, augmenting the duration of mitosis [18]. Similarly, inhibition of caspase activity in human hepatoma cells causes the failure of metaphase arrest in the presence of microtubule depolymerizing nocodazole [19]. Various apoptogenic insults can promote death at specific phases in the cell cycle indicating that the cell-cycle and apoptosis are intimately linked [20]. Apoptosis of G2/M arrested cells is a widespread observation in the literature [21]. Finally, caspase-8 is activated in EGF-stimulated primary hepatocytes and participates in mitogenic signaling as a positive regulator of G1/S transition [22]. Collectively these findings suggest that a non-death role for caspases in the mammalian cell cycle exists and, depending on the system/conditions, can modulate the fidelity of major cell cycle checkpoints.

In this study we describe a non-death role for the yeast metacaspase Yca1p. DNA content analysis of *Δyca1 vs.* wild-type shows that Yca1p accelerates G1/S and antagonizes G2/M transitions granting Yca1p-expressing cells an increased fitness in fermentive conditions. Analysis of a Yca1p catalytic inactivation mutant revealed that enzymatic activity of Yca1p is upstream of the G1/S transition phenotype. The slow growth phenotype of the *Δyca1* strain was exacerbated in the presence of the catalytic inactivation mutant resulting in an even longer procession through the S and G2/M cell cycle phases. Furthermore, genetic or chemical inhibition of Yca1p results in desensitization to nocodazole. These findings support the hypothesis that the canonical apoptotic proteins also regulate critical non-death functions in single cell organisms.

## Results and Discussion

### Ablation of *yca1* alters DNA content profiles

Mammalian caspase activity can alter cell cycle timing when observed under specific conditions. As a preliminary query to investigate a cell cycle role for Yca1p, we examined the Saccharomyces Genome Database (www.yeastgenome.org) for cell cycle specific changes in *yca1* expression [23]. The CDC15 synchronization time-course presented a marked peak of *yca1* expression at a time following the second G1/S transition post release from growth-repressive conditions (Figure S1, t = 150 min). The single surge of yca1 expression at the second G1/S transition may have been related to a phenomenon whereby a fraction of daughter cells in synchronized cultures do not separate from the mother cells until the second division after release from arrest [24]. This observation suggested a link between Yca1p and G1/S associated cell cycle dynamics. DNA content of wild-type BY4741 and Yca1p disrupted (*Δyca1*) yeast strains were quantified to examine this hypothesis. Strains were grown to mid-exponential phase and DNA content was analyzed by flow cytometry (see Figure S2 for example distributions, gating and controls). Our first experiments performed in standard YPD media exhibited no observable changes between the wild-type and mutant strains (data not shown).

**Figure 1:**
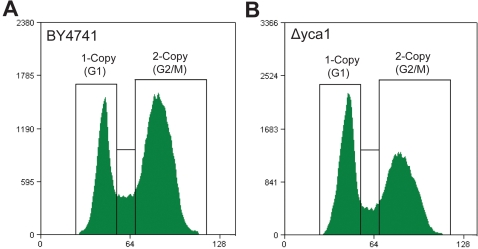
Cell cycle profiles during logarithmic growth. Representative DNA content profiles for exponentially growing (a) BY4741 and (b) *Δyca1* cells. The percentage of cells in the 1-copy and 2-copy gated regions is indicative of the percentage of cells in the respective G1 and G2/M phases of the cell cycle.

Yeast is an adaptive organism that survives in a variety of environments by modulating protein expression and activity, such as during the fermentation phase [25] where the natural habitat of the yeast is commonly the acidic surrounding of a fruit or within its confines. Given that fermentive growth may be more typical of yeast's natural environment, the experiment was repeated in acidified media. Wild-type BY4741 cells displayed no discernable change in DNA distribution between acidic and neutral media. However, *Δyca1* grown in acidic media consistently exhibited a substantial increase in the proportion of G1 cells (1-copy of DNA) and a proportionate decrease in G2/M phase cells (2-copies of DNA) when compared with wild-type controls in the same media (Fig. 1 a, b). From this we conclude that genetic ablation of *yca1* promotes a shift in the equilibrium between the 1-copy (G1) and 2-copy (G2/M) DNA populations of yeast during fermentative growth.

### Enzymatic activity of Yca1p promotes the G1/S transition and increases the robustness of log-phase yeast

Enzymatic inactivation of Yca1p via mutation of the catalytic cysteine at position 297 to alanine has been described [4]. To examine whether the enzymatic activity of Yca1p influenced cell cycle progression/equilibrium, the yca1-C297A mutant was constructed and expressed under its endogenous promoter with the selectable trophic marker leu2 in the *Δyca1* background strain (Figure S3). The resulting mutant strain (referred to henceforth as C297A) and a control strain consisting of only the selectable marker leu2 in the *yca1* locus of the same background strain (referred to as the LEU2 strain) were used. In all cell cycle experiments, the LEU2 strain behaved identically to the *Δyca1* strain indicating that the selectable marker did not influence cell cycle behavior (unpublished observation). Furthermore, reverse transcriptase PCR was used to test if modifications to *yca1* affect the expression of the genes that flank the *yca1* locus. It was observed that expression of the flanking *bfr1* and *lip5* genes were unaltered in the *Δyca1* and the C297A strains in comparison to BY4741 (Figure S4) indicating that the disruptions were specific to *yca1*.

**Figure 2:**
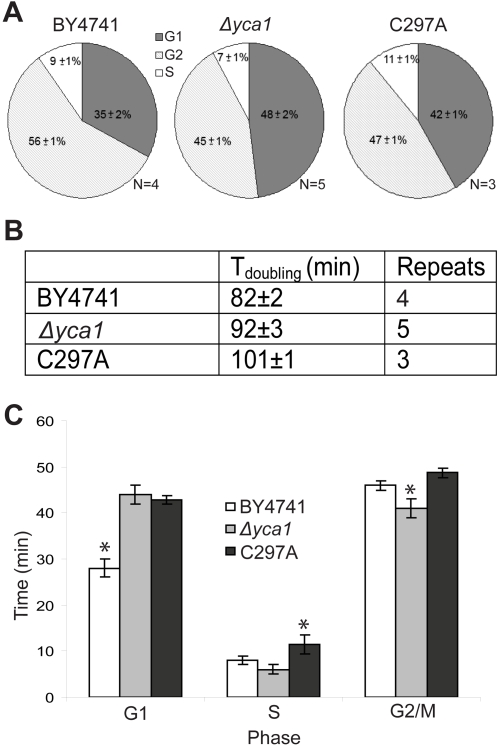
Derivation of the duration of cell cycle phases. (a) BY4741 (wild-type), *Δyca1* and C297A catalytic inactivation mutant were grown to mid-logarithmic phase in acidic YPD media. Cells were collected at regular intervals over multiple time courses and analyzed by flow cytometry. Results shown indicate the percentage of cells found in the different phases of the cell cycle. (b) Doubling time of exponentially growing cells in YPD pH 3.5 media. (c) Time spent in specific phases of cell cycle derived by multiplying the doubling time and the phase population. (* P≤0.05 heteroscedastic unpaired 2-tailed t-test, ±SEM for all).

Samples were collected at multiple time points during mid-log growth and DNA content was analyzed (see Figure S2 for example time course). The percentage of cells in each gate were quantified and averaged for all samples over the indicated time points. The averages of biological replicates were compared for the different strains and summarized as pie charts (Fig. 2 a). The doubling time of the exponentially growing cultures was then calculated based on optical density measurements. We observed that both the *Δyca1* and C297A strains had a protracted progression through the cell cycle suggesting that endogenous expression of Yca1p improves robustness of yeast cells in fermentive conditions (Fig. 2 b). The actual time spent in each phase of the cell cycle was derived by multiplying the percentage of cells in a particular phase by the observed doubling time (Fig. 2 c). One-way ANOVA confirmed significant variation of the mean within the group of different strains for each observed phase of the cell cycle (G1, S and G2/M; P<0.01). During exponential growth the time required for *Δyca1* cells to pass from G1 to S is significantly extended, passage through G2/M is accelerated and the S-phase was relatively unaffected when compared to BY4741 (Fig. 2 c). The C297A strain displayed a comparable delay through the G1/S transition just as the *Δyca1* strain suggesting that timely G1/S transition is dependent on the enzymatic activity of Yca1p. The time required for the C297A strain to pass through G2/M was comparable to that of BY4741 implying that the accelerated transition through G2/M observed for the *Δyca1* could be corrected by replacement of Yca1p without regard to its proteolytic activity. Interestingly, the C297A strain also demonstrated a protracted progression through S phase when compared to both the BY4741 and *Δyca1* strains. It is not immediately apparent how the C297A strain could alter S phase when the comparable knockout strain did not yield a similar effect. However, the substantial delay of the doubling time observed in the C297A strain suggests that YCA1-C297Ap may be acting in a dominant inhibitory fashion, interacting with the Yca1p targets and limiting their respective activity. Such an interaction could convey an illusory correction of the G2/M timing phenotype that is observed. Together, these results clearly implicate a role for the yeast metacaspase protein in a cell cycle timing function, modulating both G1/S and G2/M progression.

### Yca1p activity is required for appropriate activation of the nocodazole induced G2/M checkpoint

**Figure 3:**
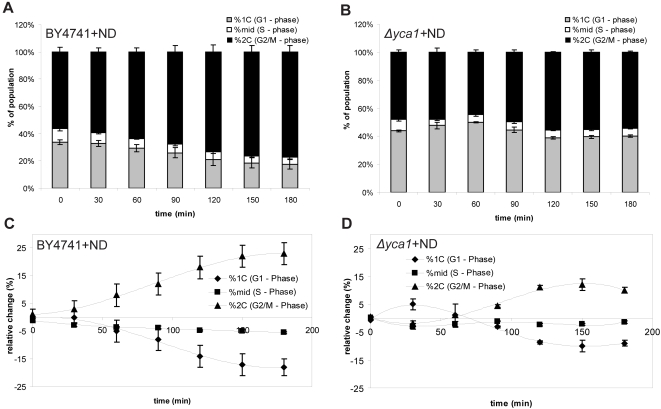
Response of cells to nocodazole. DNA content profile of wild-type (a) BY4741 and (b) *Δyca1* cells over time after treatment with 15ug/mL nocodazole. Relative change in population between nocodazole treated and untreated (c) BY4741 and (d) *Δyca1* cells. For each phase the value represents the difference between population of nocodazole treated and untreated cells (value = treated–untreated). (N = 3, ±S.E.M. for all).

Enzymatic activity of Caspase-3 in human hepatoma cells has been identified as a requirement for appropriate cell cycle arrest in response to nocodazole [19]. The observation that the G2/M timing was accelerated in the absence of the yeast caspase (Fig. 2 c) led us to hypothesize that Yca1p may perform a similar function. The following experiments were performed to test if Yca1p similarly affected the G2/M checkpoint.. When cells were treated with 15 ug/mL nocodazole in YPD media, exponentially growing BY4741 and *Δyca1* strains were predominantly arrested in G2 within 100 minutes (data not shown). BY4741 cells similarly treated in acidified media were observed to respond more slowly but did reach a primarily G2 arrested state within 180 min (Fig. 3 a). Intriguingly, the *Δyca1* cells were desensitized to the arresting agent in acidified media. Only a marginal increase in the size of the G2 population was observed (Fig. 3 b), suggesting that loss of Yca1p causes the cells to bypass the G2/M check point and continue the cell cycle proper (Fig. 3 c, d).

**Figure 4:**
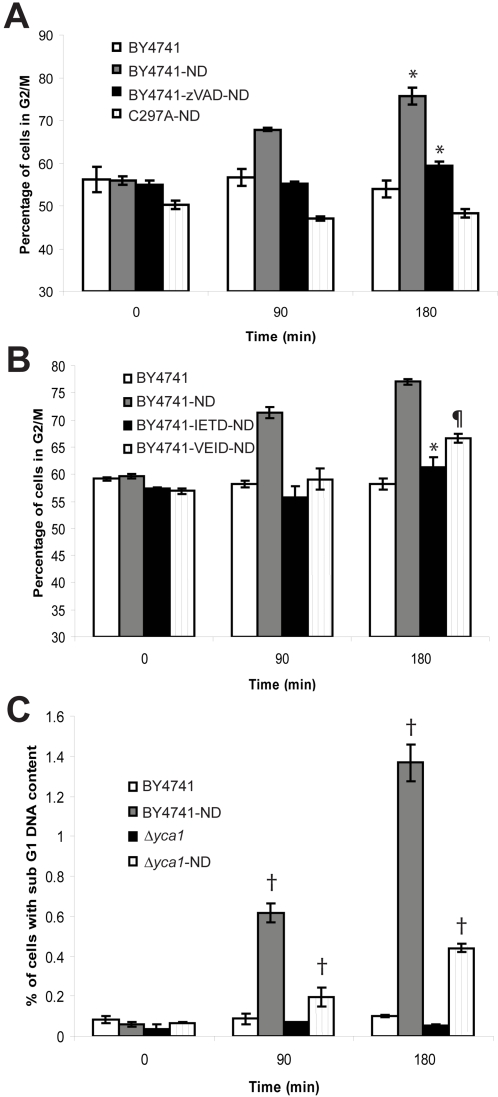
G2/M response of caspase inhibited cells. (a) G2/M content of C297A caspase catalytic inactivation mutant or wild-type BY4741 cells pre-treated for 30 minutes with 20 μM pan-caspase inhibitor z-VAD-FMK in response to 15 μg/mL nocodazole. (b) BY4741 cells pre-treated for 30 minutes with 20 μM Caspase 6 (z-VEID-FMK) and caspase 8 (z-IETD-FMK) specific peptide inhibitors before exposure to 15 μg/mL nocodazole. (c) Sub G1 population of BY4741 and *Δyca1* cells either untreated or exposed to 15 μg/mL nocodazole over time (N = 3, ±S.E.M., P≤0.05 heteroscedastic unpaired 2-tailed t-test between paired *, ¶ and † symbols; note Figure 4a is a composite of multiple experiments).

To test whether or not the G2/M defect derived from the enzymatic activity associated with Yca1p, BY4741 cells were pre-treated with the pan-caspase inhibitor z-VAD-FMK before the addition of nocodazole. The characteristic increase in the G2/M population and decrease in the G1/S population were eliminated in the presence of the caspase inhibitor. This result paralleled the response of the C297A strain to nocodazole in identical conditions, indicating that the enzymatic activity of Yca1p is required for the nocodazole response (Fig 4 a). Protease activity analogous to mammalian caspases 6 and 8 has been observed in yeast responding to H_2_O_2_ [4]. Caspase 6 and 8 specific peptide inhibitors were tested for their capacity to inhibit cell cycle arrest in response to nocodazole (Fig. 4 b). The response to both inhibitors was similar to that observed with the C297A strain and zVAD treatment, however, BY4741 cells responded with greater sensitivity to the caspase 8 inhibitor in comparison to the -6 inhibitor. To verify that the results of these experiments were not skewed by the presence of cell death in response to nocodazole [18], [26] the sub-G1 populations of both BY4741 and *Δyca1* cells were quantified (Fig. 4 c). Only a very small proportion of cells died in response to nocodazole (<2%). As such, the additional cell death in BY4741 (<1% of cells) may be explained as an apoptotic response to nocodazole activated Yca1p. We conclude that the nocodazole induced G2/M checkpoint is dependent on the enzymatic activity of Yca1p.

### The cell cycle phenotype is not caused by excessive oxidative stress

**Figure 5:**
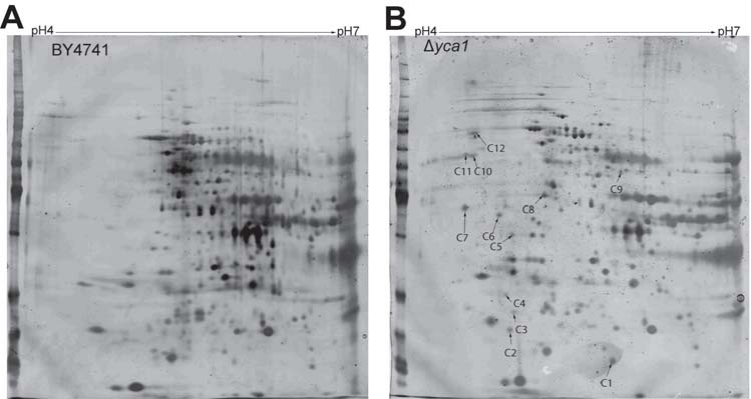
Representative 2D gels of BY4741 and *Δyca1* proteomes. (a) BY4741 and (b) *Δyca1* cells separated along pH 4-7 IPG strips. The BY4741 gels were relatively over-stained to expose yca1 inducible protein species in the *Δyca1* gel. Arrowheads depict spots that appear unique to *Δyca1* lysates. Alphanumeric designation corresponds with identities summarized in table 1.

We sought to identify proteins that could modify the G1/S checkpoint in response to *yca1* by using comparative proteomics. Whole cell lysates derived from BY4741 and *Δyca1* were resolved by 2D SDS-PAGE (Fig. 5) and examined for altered mobility in response to *yca1*ablation. We observed numerous changes to the yeast proteome between gels; particularly a number of protein species unique to *Δyca1* strain that were not apparent in protein lysates from the BY4741 strain. These spots were excised and analyzed by MS (Table 1). The list of yca1 enriched/responsive proteins included mitochondria-associated factors and proteins functioning in the oxidative stress response, the 20s proteasome and nucleotide excision repair (NER) process.

**Table 1:** MS identification of protein species induced by yca1 ablation.

Spot	Name	Score/peptides	Notes a
C1	PST2	72/2	Mitochondrial; Oxidative stress response protein induced by YAP1
C3	MRP8	52/2	Putative mitochondrial ribosomal protein
C4	PUP2	73/3	Alpha subunit of 20S proteasome
C5	LIA1	171/5	deoxyhypusine hydroxylase; Possible role in mitochondrial positioning
C6	SGT2	151/5	Similarity to human SGT which negatively regulates HSP70
C7	PEP4	155/4	Aspartate protease involved in metabolic degradation (pepsin like); important for protein turnover after oxidative damage
C8	TIF2	347/9	eIF4a homolog
C10	PYK1	90/5	Pyruvate kinase
C11	RAD23	181/4	Nucleotide excision repair; DNA damage checkpoint role normally degraded during G1/S transition
C12	PDI1	227/7	Protein disulfide isomerase involved in formation and management of scrambled protein disulfide bonds

a Notes derived from ORF descriptions available at www.yeastgenome.org.

**Figure 6:**
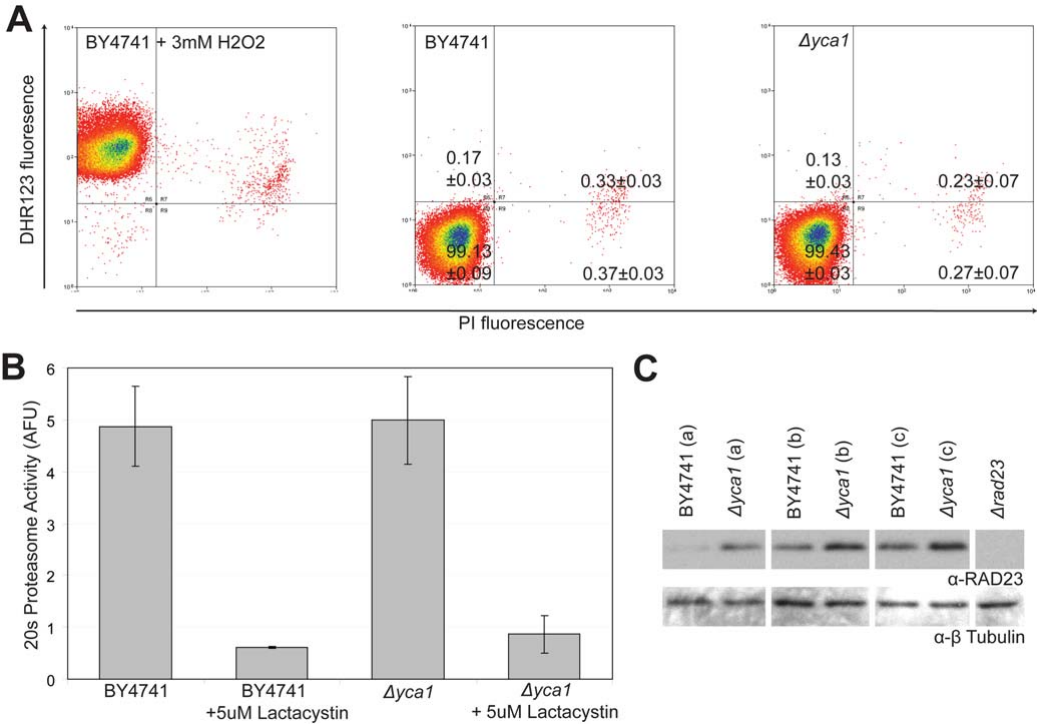
Downstream effects and oxidative stress. (a) Bivariate flow cytometry analysis of H2O2 treated BY4741, BY4741 and *Δyca1* cells dual stained with propidium iodide (PI) and ROS stain dihydrorhodamine 123 (DHR123). (N = 3, ±SEM). (b) 20s proteasome activity from BY4741 and *Δyca1* lysates (N = 3, ±SEM). (c) Western blot for 3 biological replicates of BY4741, *Δyca* and *Δrad23* (100 μg protein for each). *Δrad23* appears as a negative control. The Rad23p antibody was obtained from Santa Cruz Biotechnology (SCBT, Santa Cruz, CA), β-tubulin (Iowa State University Hybridoma Bank) was used as a loading control.

Increased oxidative damage and compensatory activity of the 20s proteasome occurs in the absence of Yca1p [7]. As such, we reasoned that altered expression of mitochondrial, stress response and DNA repair proteins might reflect the presence of oxidative DNA damage which might be responsible for the extended G1/S transition in the *Δyca1* strain. We assessed intracellular reactive oxygen species (ROS) via bivariate flow cytometry using dihydrorhodamine 123 (DHR123) as an indicator of ROS and propidium iodide (PI) as a marker of membrane integrity/viability [27]. When treated with 3mM H_2_O_2_ for 2 hours before analysis, BY4741 cells were observed to be predominantly ROS positive with a smaller percentage of non-viable PI positive cells co-staining with DHR123. Untreated exponentially growing BY4741 and *Δyca1* cells, however, were largely negative for both PI and DHR123 (Fig. 6 a). We also assessed the chymotrypsin-like activity of the 20s proteasome in BY4741 and *Δyca1* cell lysates (Fig. 6 b). We did not observe any change between the BY4741 and *Δyca1*strains which indicated that the 20s proteasome was not abnormally activated. These results suggest that the cell cycle effects and proteomic changes seen in *Δyca1* cells are not due to oxidative stress or abnormal proteasome activity.

We identified Rad23p as a potential *yca1* modulated protein. Rad23p stabilizes DNA damage repair machinery (G1 checkpoint) and is a cofactor to the ubiquitin/proteasome pathway [28]. Increased expression of Rad23p in the *Δyca1* strain was confirmed by western blot (Fig. 6 c). Rad23p is usually degraded at the G1/S transition [28]. Given the preponderance of G1 cells in the *Δyca1* strain, the increased presence of Rad23p in these cells suggests the potential activation and stabilization of the NER associated checkpoint [29]. We examined the possibility of direct interaction between Yca1p and Rad23p; however, we were unable to demonstrate any association through co-immunoprecipitation of Rad23p with the Yca1p-TAP strain or Yca1p with the Rad23p-TAP strain (unpublished observations). The lack of Rad23p cleavage products in the western blots in addition to the failure to co-immunoprecipitate suggests that the Rad23p response is secondary to Yca1p activity. However, the increase in Rad23p in the *Δyca1* strain might suggest the occurrence of alternate DNA processing events which may contribute to the G1/S phenotype.

Low-level ROS has been reported to act as a proliferative signal, stimulating G1/S progression [30], [31]. We cannot discount the possibility that retention of Rad23p and the G1/S transition phenotype result from subtle differences in ROS levels that are not detectable by our methods. However, the absence of 20s proteasome activity and ROS staining suggest that oxidative stress-associated DNA damage is not directly invoking an NER response in *Δyca1* cells. In fermentive conditions, both strains grow in a healthy manner free of excessive oxidative stress or cell death suggesting that these are not the direct causes of the cell cycle phenotype.

In conclusion, our results demonstrate a previously unrecognized role for the yeast metacaspase Yca1p in cell cycle progression. Yca1p accelerates the G1/S transition and slows the G2/M transition. In the first instance Yca1p confers a growth advantage by promoting the transition between G1 and S phase. Secondly, our results show that Yca1p antagonizes cell growth by limiting the G2/M transition. Furthermore, we observed that *Δyca1* and Yca1p inhibited cells are similarly desensitized to the nocodazole induced G2/M checkpoint. Reduced response to nocodazole has been detected in mammalian systems in which the Yca1p related enzyme caspase-3 is inhibited. Based on these observations, we speculate that the disparate ability of metacaspases to both promote and antagonize different cell cycle checkpoints may represent an early form of the proliferation/differentiation regulating activity exhibited by metazoan caspases.

## Materials and Methods

### Yeast strains and growth conditions

S. cerevisiae strains used in this study include wild-type haploid BY4741 (MATa his3Δ1 leu2Δ0 met15Δ0 ura3Δ0) and the knockout *Δyca1* strain from the BY4741 background (Open Biosystems, Huntsville, AL). Yeast cultures were grown in yeast extract peptone dextrose (YPD) medium (2% glucose, 2% Bacto peptone, and 1% yeast extract; pH adjusted to 3.5 with HCL; agar was added for solid plates to a final concentration of 2%). Overnight starter cultures grown from a single S. *cerevisiae* colony were used to inoculate large YPD cultures to a final optical density (OD660) of 0.05. These cultures were grown at 30° C with 250 RPM orbital rotation into mid-logarithmic phase at which point samples were collected as described in the *Exponential growth, caspase inhibition and cell cycle arrest* section below.

### Construction of the C297A strain

The YCA1 open reading frame in the BG1805 plasmid (Open Biosystems, Huntsville, AL.) was used as a template for site directed mutagenesis. A mutagenic fragment was generated using a mutagenic primer 5′-TGTTTGACTCTGCTCATTCGGGTAC-3′ and a reverse primer with a flanking HindIII site 5′-GGGAAGCTTCTACATAATAAATTGCAGAT-3. The 487 bp mutagenic fragment was then used in a second PCR reaction with a primer directed at the 5′ end of *yca1* 5′-GGGGGATCCATGAAGATGAGCCTCGAAGT-3′ with a flanking BamH1 site. We cloned the C297A construct into the BamH1/HindIII site of the pSSB08 plasmid; obtained by introducing two silent mutations (F: TTC to TTT ; E: GAA to GAG) to remove the EcoRI site in the Leu2 selection marker of the pRS405 backbone [32] created by Simon St-Pierre. The construct was amplified using primers containing 41-mer regions of homology to the endogenous *yca1* locus and 20-mers directed at the construct 5′-CTATTGAAAAAGCATGGCTTCGCATTAATAGGAGCCAAAAATATGAAGATGAGCCTCGAAG-3′ and 5′-CTATTGAAAAAGCATGGCTTCGCATTAATAGGAGCCAAAAATCCCCGGGCTGCAGGAATTCGA-3′. The *Δyca1* strain was transformed with the double stranded DNA product using a standard LiAC/SS carrier DNA/PEG method.

### Protein extraction

*S. cerevisiae* cells were pelleted, washed twice and resuspended in 500 μL of ice cold modified RIPA buffer (50 mM Tris-HCL, 1% NP-40, 150mM NaCl 1mM EDTA 1% Glycerol) containing protease inhibitors. 0.7g of acid washed glass beads (Sigma Aldrich, Ontario, Canada) were added and cells were lysed with a Disruptor Genie (Scientific Industries, New York, USA) at 4°C. Lysates were clarified by centrifugation at 5000 × g for 20 minutes and the soluble fraction was retained for analysis.

### Exponential growth, caspase inhibition and cell cycle arrest

For exponential growth experiments BY4741, Δ*yca1* or C297A strains were grown to OD660 of 0.4–0.5 at which point optical density was recorded and aliquots were collected at regular intervals. To inhibit endogenous caspase-like enzymatic activity cells were pretreated with 20 μM of either z-VAD-FMK, z-VEID-FMK or z-IETD-FMK (BioVision, Mountain View, CA) at an OD660 of 0.3. Cultures were allowed to grow in the presence of inhibitor for an additional 45 minutes before further treatment with 15 μg/mL nocodazole (Sigma, Saint Louis, MO) and subsequent time course sample collection. For cell cycle arrest, BY4741, Δ*yca1* or C297A cells were grown to OD660 of 0.4–0.5 before addition of 15 μg/mL nocodazole. In all cases, OD660 measurements and 1 mL aliquots were collected regularly over 3 hours. Collected samples were immediately fixed in cold 70% ethanol and stored at 4°C overnight before further analysis. Optical density at 660 nm (OD660) of liquid yeast culture was determined using an Ultrospec 2100 PRO spectrophotometer.

### DNA staining

3.5×10^6^ cells were collected from ethanol fixed samples, washed with 50 mM sodium citrate and resuspended in sodium citrate containing 0.2 mg/mL RNase A (GE Healthcare, Pittsburgh, PA). Samples were incubated at 37°C for 22 hours to promote hydrolysis of endogenous RNA. Sytox green (Molecular Probes, Burlington, Ontario) was added to a final concentration of 3.75 μM and samples were incubated in the dark at room temperature for 1 hour. Finally, samples were pelleted and resuspended in PBS before analysis by flow cytometry. DakoCytomation Summit version 4.0 software was used to gate and quantify cells after flow acquisition (see Figure S2 for example gating).

### Flow cytometry

Flow cytometry data acquisition was performed with a Coulter MoFlo (Fullerton, California, USA), all samples were excited by a 488 nm Spectra-Physics Argon laser, Sytox green dye and DHR123 fluorescence was detected with a 530/40 bandpass filter. PI was detected with a 630/30 bandpass filter. 50000 cells per sample were counted. Dual compensation was performed to remove signal bleed between the PI and DHR123 channels. Flow data was analyzed with DakoCytomation Summit version 4.0 software.

### 2D gel electrophoresis

Samples were diluted in isoelectric focusing buffer (7M urea, 2M thiourea, 4% CHAPS, 1% DTT) and absorbed into 17 cm ReadyStrip™ IPG strips (Biorad) following the manufacturer's directions. Isoelectric focusing was performed on a Protean IEF Cell (Biorad) with the following program: 200 V for 1 hour, 500 V for 1 hour, 5000 V ramp for 5 hours, 5000 V for 80000 VH. For the second dimension, IPG strips were overlaid onto 10% SDS-PAGE gels and electrophoresed in a Protean II apparatus (Biorad).

### Mass spectrometry

Silver stain and in-gel digest were performed using a standard method [33]. Sample cleanup was performed using ZipTip (Millipore). Tandem mass spectra were collected on an Applied Biosystems/SciEX QSTAR XL with oMALDI2 ion source. Spectra were analyzed using Mascot 2.0 (Matrix Science, UK) and the NCBI non-redundant protein sequence database (version 20070306). MS/MS tolerance was ±0.4 Da.

### 20s Proteasome activity assay

50 μg of clarified whole cell extract was analyzed using the Proteasome Activity Kit (Chemicon, California, USA). Fluorescent AMC liberated from the 20s proteasome substrate suc-LLVY-AMC was detected for 90 minutes at 37°C as per instructions on a Fluoroskan Ascent FL microplate fluorometer (Thermo Scientific, USA) with excitation at 380nm and emission at 460 nm.

### Supplementary Information

Figure S1 shows expression pattern of Yca1p following release from CDC15 synchronization. Figure S2 displays G1 and G2/M gating controls in addition to example gating as used for time course DNA profiles. Figure S3 depicts the verification of the C297A strain. Figure S4 demonstrates the invariability of the genes immediately flanking the YOR197w open reading frame.

## Supporting Information

Figure S1 Expression of yca1 after cdc15 synchronization. Adaptation of data from Spellman et al. (Mol Biol Cell 9, 3273-97 1998) depicting expression of YCA1 after CDC15 synchronization. Arrows denote the first observation of new buds. Peak expression after 150 minutes release coincides with second appearance of small buds indicative of the G1/S transition in budding yeast.
(0.10 MB DOC)

Figure S2 DNA staining and flow cytometry gates. (a) 63× phase contrast/FITC microscope merge of sytox green stained yeast cells arrested with nocodazole. Microscopy was performed on an Axioplan 2 compound microscope fitted with a Zeiss Axiocam. Fixed cells were suspended in 50mM sodium citrate, slide mounted and viewed at 63× magnification with a PlanApochromat 1.4 NA objective lens at 22°C. Phase contrast and FITC channel images were merged using Axiovision 3.1 software (Carl Zeiss, Toronto Canada). (b) Control DNA content profile demonstrating gating used to quantify cell cycle populations for nocodazole treated BY4741 cells. The histogram abscissa represents relative fluorescent intensity of fixed cells incubated with the nucleic acid stain Sytox green. (c,d) Example of gating used to identify G1 and G2 peaks. Cells were arrested at the G1/S transition with (c) the ribonucleotide reductase inhibitor Hydroxyurea or in the G2/M phase with (d) Nocodazole. (e) Example time response (in minutes) and gating of wild type BY4741 cells to 15 μg/mL nocodazole. The montage demonstrates a progressive decay of the G1 population concurrent with an enlargement of the G2 population consistent with G2 arrest.
(0.60 MB DOC)

Figure S3 Verification of integration, deletion and expression. Integration of the C297A construct into the Δyca1 background strain. (a,b) The location of integration and expression of integrants were verified using A–B and C–D primers designed for the systematic deletion project (http://www-sequence.stanford.edu/group/yeast_deletion_project/confirmation.html) predicted 663 bp and 2.7 kb fragments were observed. (c) Reverse transcriptase PCR confirming the presence of the C297A transcript. ADH3 was used as a loading control.
(0.42 MB DOC)

Figure S4 Expression of yca1 flanking genes. a) Region of chromosome XV depicting genes flanking yca1. b) Reverse transcriptase PCR of flanking genes demonstrates invariable expression in the Δyca1 and C297A strains when compared to the congenic BY4741 background strain. ADH3 was used as a loading control.
(0.20 MB DOC)
