# Supplementary material for: A Non-Death Role of the Yeast Metacaspase: Yca1p Alters Cell Cycle Dynamics
Source: PLoS One. 2008 Aug 13;3(8):e2956. doi: 10.1371/journal.pone.0002956 (PMC2493032; doi:10.1371/journal.pone.0002956)
Supplement: Figure S1 — Expression of yca1 after cdc15 synchronization. Adaptation of data from Spellman et al. (Mol Biol Cell 9, 3273-97 1998) depicting expression of YCA1 after CDC15 synchronization. Arrows denote the first observation of new buds. Peak expression after 150 minutes release coincides with second appearance of small buds indicative of the G1/S transition in budding yeast. (0.10 MB DOC) [file pone.0002956.s001.doc]

**Figure S1. Expression of yca1 after cdc15 synchronization.** Adaptation of data from Spellman et al. (Mol Biol Cell 9, 3273-97 1998) depicting expression of YCA1 after CDC15 synchronization. Arrows denote the first observation of new buds. Peak expression after 150 minutes release coincides with second appearance of small buds indicative of the G1/S transition in budding yeast.
