# Supplementary material for: A Non-Death Role of the Yeast Metacaspase: Yca1p Alters Cell Cycle Dynamics
Source: PLoS One. 2008 Aug 13;3(8):e2956. doi: 10.1371/journal.pone.0002956 (PMC2493032; doi:10.1371/journal.pone.0002956)
Supplement: Figure S2 — DNA staining and flow cytometry gates. (a) 63× phase contrast/FITC microscope merge of sytox green stained yeast cells arrested with nocodazole. Microscopy was performed on an Axioplan 2 compound microscope fitted with a Zeiss Axiocam. Fixed cells were suspended in 50mM sodium citrate, slide mounted and viewed at 63× magnification with a PlanApochromat 1.4 NA objective lens at 22°C. Phase contrast and FITC channel images were merged using Axiovision 3.1 software (Carl Zeiss, Toronto Canada). (b) Control DNA content profile demonstrating gating used to quantify cell cycle populations for nocodazole treated BY4741 cells. The histogram abscissa represents relative fluorescent intensity of fixed cells incubated with the nucleic acid stain Sytox green. (c,d) Example of gating used to identify G1 and G2 peaks. Cells were arrested at the G1/S transition with (c) the ribonucleotide reductase inhibitor Hydroxyurea or in the G2/M phase with (d) Nocodazole. (e) Example time response (in minutes) and gating of wild type BY4741 cells to 15 μg/mL nocodazole. The montage demonstrates a progressive decay of the G1 population concurrent with an enlargement of the G2 population consistent with G2 arrest. (0.60 MB DOC) [file pone.0002956.s002.doc]

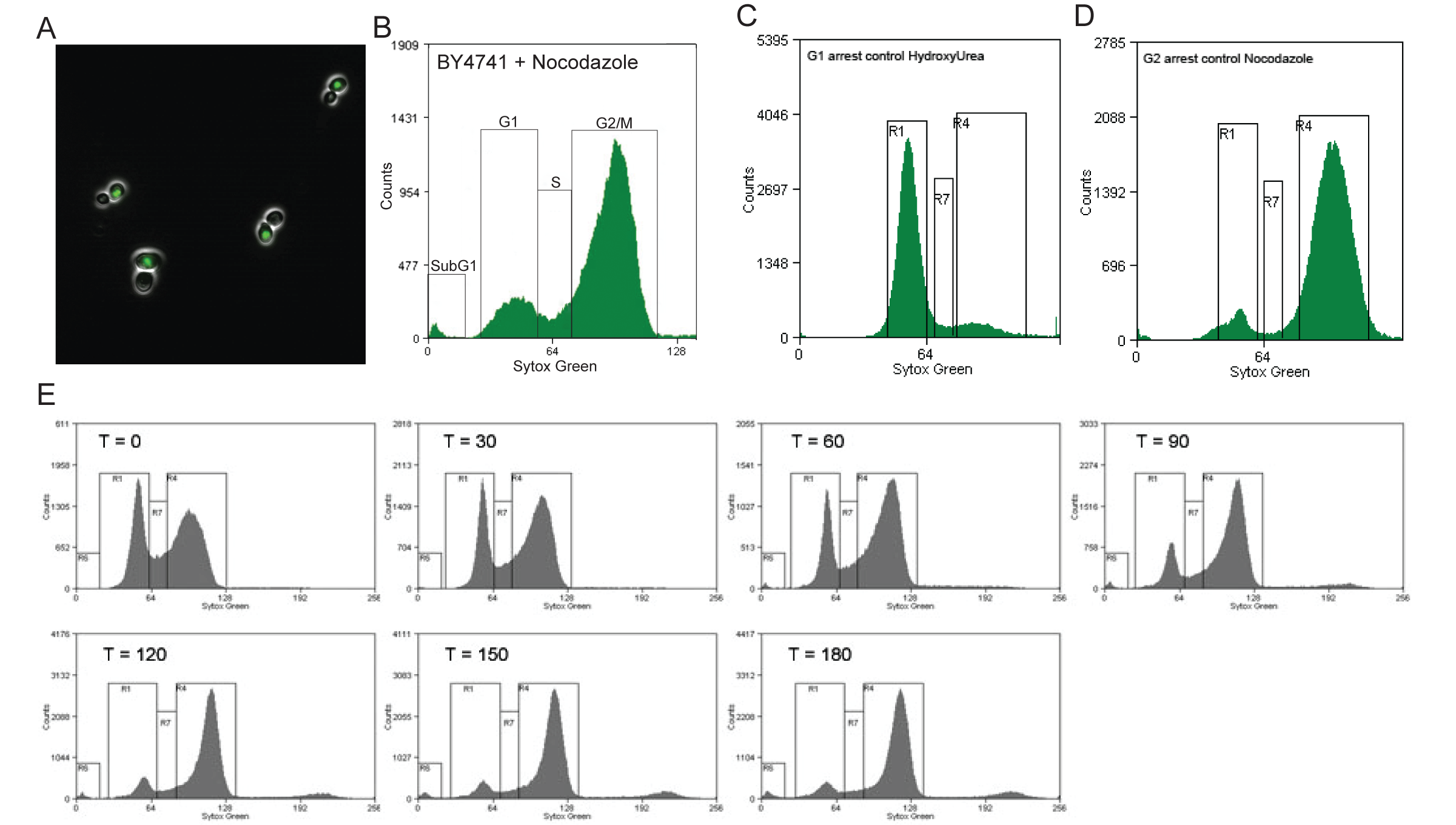


**Figure S2.** **DNA staining and flow cytometry gates (a)** 63x phase contrast/FITC microscope merge of sytox green stained yeast cells arrested with nocodazole. Microscopy was performed on an Axioplan 2 compound microscope fitted with a Zeiss Axiocam. Fixed cells were suspended in 50mM sodium citrate, slide mounted and viewed at 63x magnification with a PlanApochromat 1.4 NA objective lens at 22˚C. Phase contrast and FITC channel images were merged using Axiovision 3.1 software (Carl Zeiss, Toronto Canada). **(b)** Control DNA content profile demonstrating gating used to quantify cell cycle populations for nocodazole treated BY4741 cells. The histogram abscissa represents relative fluorescent intensity of fixed cells incubated with the nucleic acid stain Sytox green. **(c,d)** Example of gating used to identify G1 and G2 peaks. Cells were arrested at the G1/S transition with **(c)** the ribonucleotide reductase inhibitor Hydroxyurea or in the G2/M phase with **(d)** Nocodazole. **(e)** Example time response (in minutes) and gating of wild type BY4741 cells to 15 μg/mL nocodazole. The montage demonstrates a progressive decay of the G1 population concurrent with an enlargement of the G2 population consistent with G2 arrest.
