# Supplementary material for: A Non-Death Role of the Yeast Metacaspase: Yca1p Alters Cell Cycle Dynamics
Source: PLoS One. 2008 Aug 13;3(8):e2956. doi: 10.1371/journal.pone.0002956 (PMC2493032; doi:10.1371/journal.pone.0002956)
Supplement: Figure S3 — Verification of integration, deletion and expression. Integration of the C297A construct into the Δyca1 background strain. (a,b) The location of integration and expression of integrants were verified using A–B and C–D primers designed for the systematic deletion project (http://www-sequence.stanford.edu/group/yeast_deletion_project/confirmation.html) predicted 663 bp and 2.7 kb fragments were observed. (c) Reverse transcriptase PCR confirming the presence of the C297A transcript. ADH3 was used as a loading control. (0.42 MB DOC) [file pone.0002956.s003.doc]

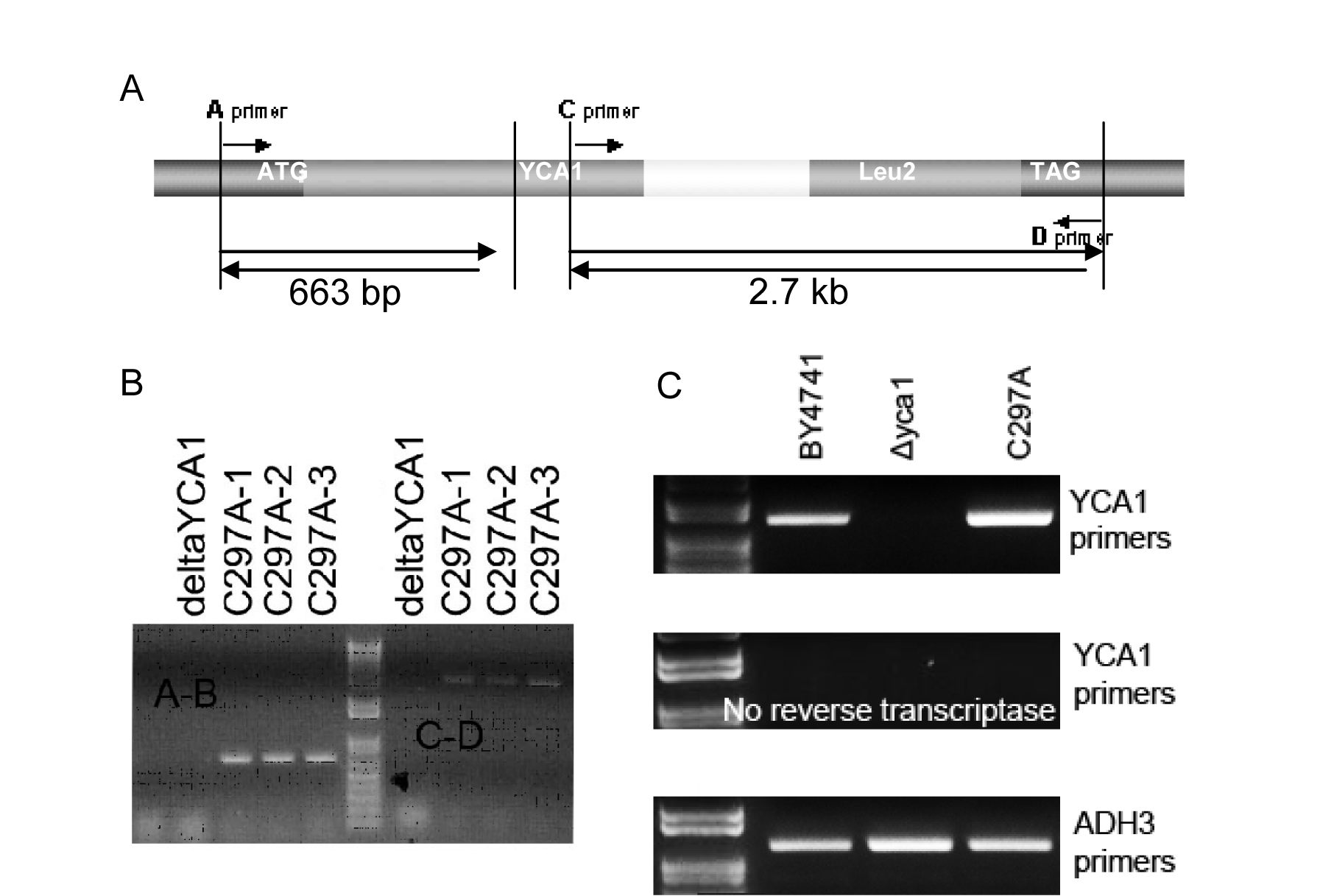


**Figure S3. Verification of integration, deletion and expression.** Integration of the C297A construct into the *Δyca1* background strain. **(a,b)** The location of integration and expression of integrants were verified using A-B and C-D primers designed for the systematic deletion project (http://www-sequence.stanford.edu/group/yeast_deletion_project/confirmation.html) predicted 663 bp and 2.7 kb fragments were observed. **(c)** Reverse transcriptase PCR confirming the presence of the C297A transcript. ADH3 was used as a loading control.
