# Supplementary material for: A Non-Death Role of the Yeast Metacaspase: Yca1p Alters Cell Cycle Dynamics
Source: PLoS One. 2008 Aug 13;3(8):e2956. doi: 10.1371/journal.pone.0002956 (PMC2493032; doi:10.1371/journal.pone.0002956)
Supplement: Figure S4 — Expression of yca1 flanking genes. a) Region of chromosome XV depicting genes flanking yca1. b) Reverse transcriptase PCR of flanking genes demonstrates invariable expression in the Δyca1 and C297A strains when compared to the congenic BY4741 background strain. ADH3 was used as a loading control. (0.20 MB DOC) [file pone.0002956.s004.doc]

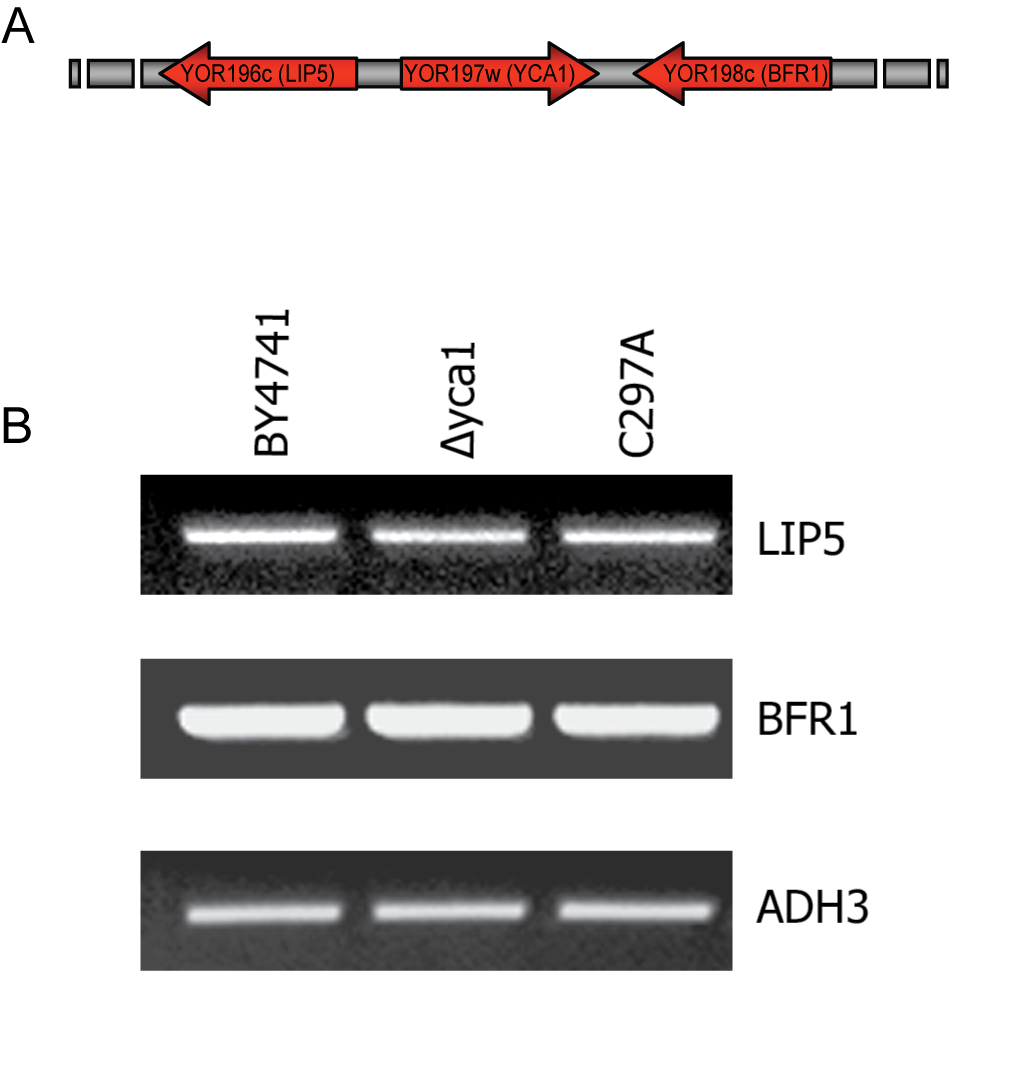


**Figure S4. Expression of *yca1* flanking genes. a)** Region of chromosome XV depicting genes flanking yca1. **b)** Reverse transcriptase PCR of flanking genes demonstrates invariable expression in the Δyca1 and C297A strains when compared to the congenic BY4741 background strain. ADH3 was used as a loading control.
